# List of Members Who Paid Assess. No. 4

**Published:** 1888-01

**Authors:** 


[Dr. Kilpatrick was holder of certificate No. 46. He joined the Association in April, 1884, and had paid $4 in dues, and three assessments of $1 each, a total of $7. Ed.]

List of members Physicians' Mutual Benefit Association who paid the assessment No. 4:

F. E. Daniel, Austin.
M. A. Taylor, "
J. W. McLaughlin, Austin.
R. M. Swearingen, "
J. M. Litten, "
W. A. Morris, "
T. J. Bennett, "
A. N. Denton, "
W. P. Burts, Ft. Worth.
J. T. Field, "
A. S. Wolf, Brownsville.
H. C. Ghent, Belton.
S. W. Sholars, Orange.
Geo. Cupples, San Antonio.
F. Herff, "
D. V. Spring, Lyons.
R. H. Day, Baton Rouge, La.
J. B. Duchiene, " "
J. W. Dupree, " "
S. A. Towsey, Galveston.
J. P. Coffey, Plano.
T. M. Matthews, Athens.
M. D. Sterrett, Grand Bluff.
E. S. Tidwell, Elmo.
A. M. Hill, Hill's Prairie.
J. M. Ross, Belton.
C. C. Francis, Cleburne.
T. C. Osborn, "
J. M, Keating, "
H. H. Thorp, Liberty Hill.
J. A. Throckmorton, Houston.
J. M. Hons, Burton.
J. R. Scales, Henderson.
F. T. Paine, Comanche.
H. W. Brown, Waco.
W. H. Wilkes, "
R. G. Williams, Whitney.
S. F. Styles, Independence.
M. M. Myers, Merle.
C. E. R. King, San Antonio.
B. E. Hadra, Austin.
W. M. Powell, Albany.
L. H. Hardy, Paige.
C. O. Weller, Austin.
T. J. McFarland, Edna.
J. W. Daniel, Houston.
Jno. Inabnit, Terrell.
F. A. Tompkins, Sandy Point.
A. D. Paulus, Flatonia.
F. W. Kaiser, "
J. T. Webb, Terrell.
Y. D. Harrington, Los Angelos, Cal.
G. W. Gray, Terrell.
J. M. Ball, New Boston.
R. W. Reid, Texarkana.
W. T. Strain, McCoy City.
J. K. Frazer, Rusk.
W. D. Boyd, Orilla
A. Wadgymar, Corrizo Springs.
W. Gluver, Lovelady.
J. W. Fennell, Seguin.
E. P. Becton, Sulphur Springs.
Q. A. Shuford, Tyler,
T. S. Burke, Corpus Christi.
J. H. Bowers, Columbus.
A. L. Patton, Mineola.

A. N. Perkins. Sabine Pass.
J. E. Mayfield, Nacogdoches.
F. C. Ford "
T. J. Murph, "
Q. C. Smith, Austin.
A. D. Tinsley, Henderson.
J. H. McClintock. Houston.
Sam. J. Fox, Plantersville.
W. C. Jameson, Rusk.
F. R. Martin, Kyle.
R. S. Gregg, Manor.
W. T. Richmond, "
J. M. Lewis, Mexia.
F. M. Wilson, Kosse.
J. H. Evans, Alto.
J. T. Y. Jameson, Rusk.
W. A. McGee. Uvalde.
M. F. Wakefield, Edom.
R. A. Taylor, Milford.
J. B. Stinson, Sherman.
J. E. Kirkley, Belville.
J. M. Fry, Elmo.
A. D. Evans, Floresville.
J. A. White, Bartlett.
M. O. Wright, "
A. J. Folsom, McGregor.
C. Richardson, Bushy.
T. J. Bell, Tyler.
E. R. W. McCrary, Hopewell.
J. A. Southworth, Corsicana.
H. F. Witherspoon, "
Ebenezer Jones, Tyler.
J. G. Daniels, Gilmer.
J. D. Bass, Pittsburg.
T. N. Pitts, "
H. F. Ford, Gilmer.
L. D. Hill, Webberville.
R. H. Harrison, sr., Columbus.
A. Patton, Mineola.
Jos. Greer, Van Alstyne.
M. R. Denman, St. Elmo.
R. Leming, Mossville.
R. P. Talley, Belton.
W. H. Lancaster, Moulton.
C. M. Harrison, El Paso.
J. A. Denson, Granger.
W. J. Dupree, Mineola.
C. Sams, Taylor.
J. H. T. King, Laredo.
T. J. Watlington, Texarkana.
T. G. May, Palmer.
J. Getzwiller, Goliad.
S. A. Stone, Richmond.
T. J. Foster, Alexander,
J. T. Musick, Pittsburg.
A. W. Crawford, Midlothian.
Z. T. Bundy, "
R. P. Davies, Petty.
J. W. Hamilton, Webberville.
W. M. Burger, Montana Territory.
T. W. Styles, Paige.
W. A. Taylor, Elgin.
A. C. Walker, Ft. Worth.
G. W. Cain, Elgin.
J. G. Barbee, Kyle.
C. Bullittt, Orange.
H. S. Robertson, Peytonville, Ark.
E. G. Nicholson, Del Rio.
G. J. Stearnes, San Antonio.
J. F. W. Metzler, Rose Hill.
J. M. Brittain, Jacksonville.
J. A. Abney, Lufkin.
J. D. Beck, Mason.
H. M. Burroughs, Dalby.
D. M. Vawter, Carthage.
A. McDaniel, Columbus.

R. H. Harrison, jr., Columbus.
L. T. Maddox. Birdston.
Jas. Byars, Columbus.
Todd Robinson, Alleytown.
J. S. Bruce, Eagle Lake.
S. F. Starley, Tyler.

Total, 148; 9 forfeited.

Since the date of Dr. Kilpatrick's death, Sept. 19, 1887, twelve members have joined, making, with the 157 at that date, less the nine who forfeited, a membership of 160 at the date of Dr. Starley's death, Dec. 19, 1887, less the one death, and two members who have joined since that date. There are, therefore, 157 liable to the assessment for Dr. Starley's family—provided none forfeit for non payment of dues for 1888, now past due.

